# Evaluation of a gaze-controlled vision enhancement system for reading in visually impaired people

**DOI:** 10.1371/journal.pone.0174910

**Published:** 2017-04-05

**Authors:** Carlos Aguilar, Eric Castet

**Affiliations:** 1 BCL, Nice Sophia Antipolis Univ, CNRS, Nice, France; 2 LPC, Aix Marseille Univ, CNRS, Marseille, France; Universidade do Minho, PORTUGAL

## Abstract

People with low vision, especially those with Central Field Loss (CFL), need magnification to read. The flexibility of Electronic Vision Enhancement Systems (EVES) offers several ways of magnifying text. Due to the restricted field of view of EVES, the need for magnification is conflicting with the need to navigate through text (panning). We have developed and implemented a real-time gaze-controlled system whose goal is to optimize the possibility of magnifying a portion of text while maintaining global viewing of the other portions of the text (condition 1). Two other conditions were implemented that mimicked commercially available advanced systems known as CCTV (closed-circuit television systems)—conditions 2 and 3. In these two conditions, magnification was uniformly applied to the whole text without any possibility to specifically select a region of interest. The three conditions were implemented on the same computer to remove differences that might have been induced by dissimilar equipment. A gaze-contingent artificial 10° scotoma (a mask continuously displayed in real time on the screen at the gaze location) was used in the three conditions in order to simulate macular degeneration. Ten healthy subjects with a gaze-contingent scotoma read aloud sentences from a French newspaper in nine experimental one-hour sessions. Reading speed was measured and constituted the main dependent variable to compare the three conditions. All subjects were able to use condition 1 and they found it slightly more comfortable to use than condition 2 (and similar to condition 3). Importantly, reading speed results did not show any significant difference between the three systems. In addition, learning curves were similar in the three conditions. This proof of concept study suggests that the principles underlying the gaze-controlled enhanced system might be further developed and fruitfully incorporated in different kinds of EVES for low vision reading.

## 1 - Introduction

Age-related Macular Degeneration (AMD), a severe maculopathy, is the most common cause of low vision and often causes dramatic Central Field Loss (CFL) among elderly people who are therefore constrained to use eccentric viewing [1]. This scotoma in the center of the visual field dramatically disrupts reading performance [2,3]. Reading speed is a key performance measure that has been extensively investigated in low vision reading [4–13]. One major goal of people with CFL is to improve their ability to read text [14]. To achieve this goal, magnifying optical and/or electronic aids are commonly prescribed to help maintain the ability to read [15–17], even though magnification never restores reading speed to the level observed without CFL [18].

Electronic Vision Enhancement Systems (EVES) have a great potential to improve perceptual performance of low vision patients [19,20]: in addition to magnification, they can provide many kinds of visual enhancements [21]. A recent survey indicated that most low vision patients express an interest in image processing technology that could be implemented for television viewing and for computer use [22]. Common commercially-available EVES are closed-circuit televisions (CCTVs). One important limitation of EVES is a reduced field of view with high levels of magnification, an issue which has received considerable attention in the context of low vision reading [23–25]. When reading highly magnified text on a screen, only a portion of the line of text is visible at any moment. A technique, called page navigation, must thus be used to reveal successive parts of the text on the display screen. With a standard stand-mounted CCTV, a page of printed text lies on a movable x-y platform under a video camera. The part of the page that lies in the camera's field of view is displayed on a monitor and can be magnified (whole field magnification with a zooming center coinciding with the monitor center). To navigate through the page, the reader must move the platform either in the x direction to read along the line or in the y direction to jump from one line to the other. The subject can adjust the magnification level with a knob placed somewhere on the device. In clinical practice, patients are usually advised during a preliminary phase to adjust this knob until they feel comfortable (during this phase they do not move the platform). Once this level is found, patients are further advised to keep it constant so that their hands can be used to move the platform. Even when patients are allowed to change magnification during reading, only a very small minority uses this possibility (Burggraaff, personal communication): this was observed during a large-scale investigation of the effects of training when using standard CCTVs in visually impaired adults [26,27].

However, it is likely that keeping the magnification level constant is not the most efficient procedure. This was made clear by Culham et al. in 2004 (p.288): "Variable magnification at a fixed viewing distance should be a valuable feature in a low vision device. The facility of keeping magnification low when appropriate, allows a wider field of view, but the option of providing the patient with an acuity reserve [17] when required should be beneficial."[28]. We agree with this important suggestion. A wide field of view is for instance important when patients make long backward saccades to re-read several words. In contrast, at some other instants, identification of some words might be so difficult that a high level of magnification is necessary [29]. It should be noted here that the need to alternate between global information and more local information does not imply that magnification should be applied to the whole image. This might instead be achieved by magnifying only the local portions of the visual scene that the subject wants to identify. For this purpose, using gaze as a pointer that controls the regions of interest to be magnified seems an interesting possibility. Researchers in the field of human-computer interactions have emphasized the importance of eye-tracking [30], and gaze-controlled magnification systems have already been designed and tested for normally-sighted subjects [31–33]. A typical application of this line of research is to offer an efficient and rapid way to alternate between the global view of a large complex image (say the map of a town) and a local magnified view of details (such as streets). In the present study, we investigated whether this gaze-controlled magnification approach, which has been found successful for foveal vision, might be specifically applied to reading with CFL. We also compared this gaze-controlled “intelligent” local zooming system with two CCTV-like conditions that allowed user-friendly changes of magnification levels.

## 2 - Methods

### 2.1 – Methods for designing the gaze-controlled visual aid (condition 1)

**Fig 1:**
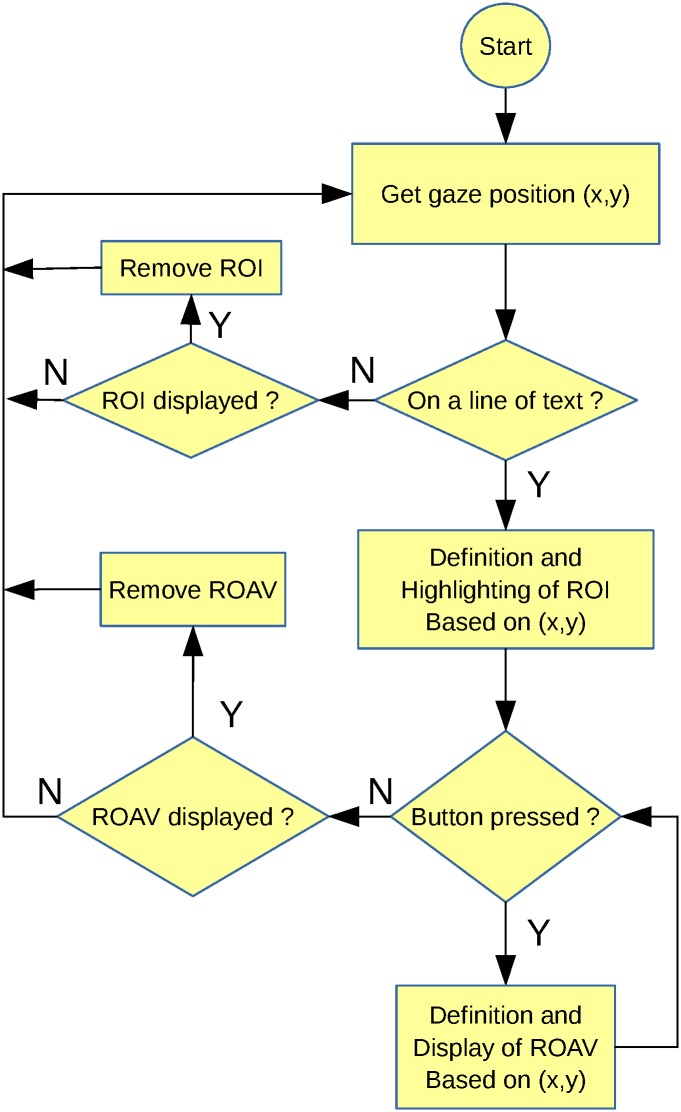
Flowchart of the key principles underlying the gaze-controlled visual aid (condition 1).

The gaze-controlled zooming aid was developed as part of a project funded by Essilor International. This project investigates the feasibility and the potential benefits for low vision patients of using an integrated system combining gaze-control functions with see-through glasses. The general principle of our system is to use gaze direction to define a region of interest (ROI)–visible on the screen—that can subsequently be visually enhanced (see flowchart in Fig 1). We refer to this approach as gaze-controlled "local enhancement". Subjects, if and only if they wish, can trigger this local enhancement by a manual control. In the present work, visual enhancement was a magnification (“zooming”) of the ROI (other more sophisticated kinds of enhancement might also be considered in the future). Several types of eye-controlled zooming interfaces with an additional manual activation have been developed for normally-sighted subjects in the field of human-machine interface [eg. 32,33]. These developments are particularly promising in contexts in which traditional mouse and keyboard input may not be available or even feasible, as for the interaction with public displays, multi-display setups, large-sized TV sets or see-through glasses. In the long term, see-through glasses equipped with eye-trackers and appropriate image processing techniques, should be able to allow many types of gaze-based interaction with distant real stimuli or displays. Such systems are heavily investigated in the medical field in order to provide 3-D medical visualization techniques based on augmented reality [34,35]. Overall, it seems that these developments also have a great potential for low vision patients [36] and have therefore been used to guide our work.

**Fig 2:**
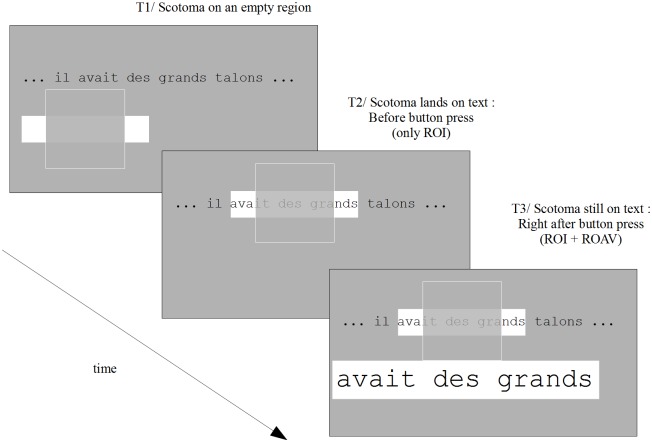
Schematic illustration of the gaze-contingent visual aid across time at three successive instants. Note that the gray rectangle (one for each instant) shows only a small portion of the screen. Note also that only one line of text is presented here for clarity. T1/ the scotoma (outlined square) is over an empty region: the Region Of Interest (ROI) thus appears as a rectangle filled with white. The width of the ROI is constant: 16 characters. T2/ the scotoma's center is on a line of text: the ROI now highlights 16 characters whose middle location corresponds to scotoma’s center. Subjects have to decide whether or not they want to trigger an enhancement of the ROI by pressing a button. T3/ After a button press, the Region Of Augmented Vision (ROAV) appears below the scotoma while the initial ROI remains highlighted. As long as the button is pressed, the whole display remains the same and subjects can explore the ROAV with eye movements (see Fig 3). After button release (not shown here), the ROAV and its corresponding ROI disappear and a new gaze-contingent ROI is displayed based on the new gaze location. In this figure and the following, the artificial scotoma is transparent for visual clarity but it was opaque in the actual experiments.

The main features of our gaze-contingent system are the following. The first feature concerns the way by which subjects choose the region that they want to magnify. This is achieved by positioning the scotoma on a line of text at the approximate location that the subject wants to magnify. When the scotoma's center location lies on a given line, a fixed-width portion (in letters) of this line of text extending to the left and right of the scotoma is highlighted (Fig 2T2). This highlighted area is the ROI: it provides a visual feedback to the subject and indicates that this area (including the part hidden by the scotoma) can be magnified and displaced, if needed, by a button press. After a button press, a new region, that we call the Region Of Augmented Vision (ROAV), appears below the scotoma (Fig 2T3): this new region is a magnified and displaced version of the ROI.

**Fig 3:**
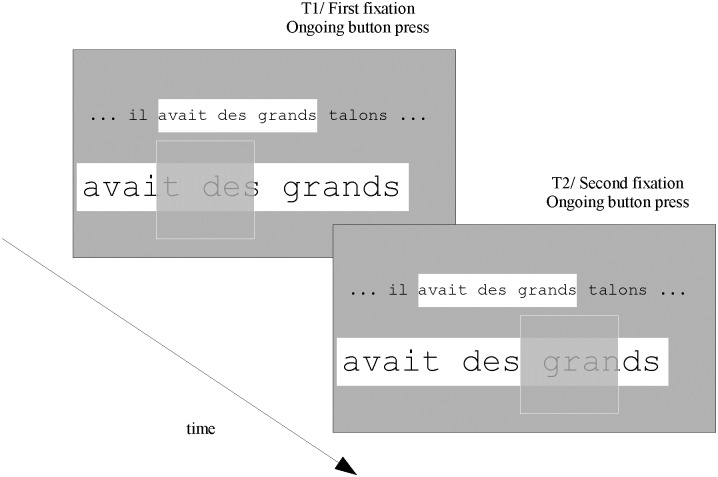
Potential continuation of Fig 2: visual exploration of the Region Of Augmented Vision (ROAV) with two successive ocular fixations. As long as the ROAV is displayed (i.e. as long as the button is pressed), subjects can make ocular fixations on different parts of the ROAV.

After being displayed, the ROAV remains on the screen as long as the button is pressed, thus allowing the subject to make ocular saccades to explore the ROAV (Fig 3). When releasing the button, the ROAV and its corresponding ROI disappear; then a new ROI appears with its position defined by the current gaze location. In the present work, the button used for these controls was the central button of a 5-button response box (RESPONSEPixx Handheld—VPixx Technologies). The top and bottom buttons of the box were used to move the text in the vertical direction (only when the ROAV was not displayed).

To implement the general principles described above, the following parameters are used. The ROI center coincides with the scotoma center. The ROI width is 16 characters so that it appears as two 3-character wide rectangles on each side of the 10° scotoma. The interline size determines the ROI height. When the ROI lies on a character-free region of the background, it appears as a white rectangle. When the ROI contained characters, it appears as a white rectangle containing black characters (highlighting). Character's size within the ROAV is twice as big as that within the ROI.

**Fig 4:**
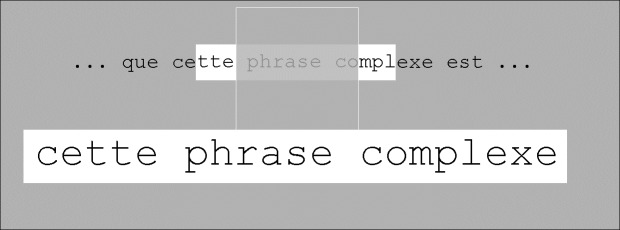
Example of an ROI whose left and right borders are located within words ("cette" and "complexe"). In this particular example, each of these 2 words has more than 50% of its letters within the ROI so that these words are included within the ROAV. Note that the large gray rectangle shows only a small portion of the monitor’s screen.

The example shown in Fig 2(T2) and 2(T3) does not represent the most common situation. This is a simple case where the ROI contains only full words, so that the mapping between the ROI and the ROAV is straightforward: the ROI contains exactly three words and has been transformed into a 3-word ROAV. In most cases, however, the situation is more complex as the left and right ROI borders are often located within words (Fig 4). In these cases, different rules determining the ROI/ROAV mapping are possible. The point here is that the main goal of these mapping rules is to produce an ROAV containing entire words, i.e. the magnification process never produces "mutilated" words. In contrast, when using a standard lens (optical or electronic), such a magnification-induced mutilation occurs most of the time depending on the lens position with respect to the word (and to the screen border) and on the ratio between word size and lens size (Note that this feature of our system can be generalized to natural images where image processing techniques could be used to segment faces or objects from their background, so that any of these faces or objects would always appear in its entirety once magnified).

**Fig 5:**
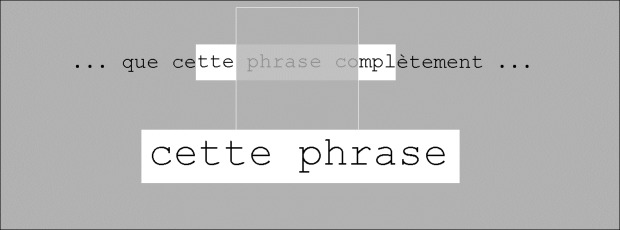
Example of an ROI whose left and right borders are located within words ("cette" and "complètement"). Here, in contrast with the example shown in Fig 4, less than 50% of the rightmost word's letters are contained within the ROI, so that this word ("complètement") is not included within the ROAV. Note that the large gray rectangle shows only a small portion of the monitor’s screen.

Thus, our algorithm aims at injecting some smartness into the “zooming” process: while the ROI width is constant (16 characters), the ROAV width is not constant because there is no one-to-one mapping between the ROI letters and the ROAV letters. To determine the characters contained within the ROI that will be displayed within the ROAV, the following conditional rules are used. As already explained, the simplest case is represented in Fig 2 where the ROI left border is just before the first letter of the ROI leftmost word ("avait") and the ROI right border is just after the last letter of the ROI rightmost word ("grands"). In this case, the ROAV content is the same as the ROI content, i.e. the ROAV contains the same 16 characters as the ROI. However, in most cases, such a spatial alignment does not occur (as illustrated above in Fig 4) and the following rule is then used. The leftmost and rightmost ROI words are only included in the ROAV if more than 50% of their letters (for each word) are contained within the ROI. This is what happens in Fig 4 where the words "cette" and "complexe" are displayed in the ROAV although they were not entirely contained within the ROI (i.e. the width of the ROAV is larger than the 16-character width of the ROI). In contrast, Fig 5 illustrates a different case where the rightmost word ("complètement"), although bridging across the ROI border, is not included within the ROAV. This is because less than 50% of its letters are contained within the ROI. The mapping rule thus creates an ROAV that contains only two full words, (i.e. a smaller width in letters than the ROI width).

**Fig 6:**
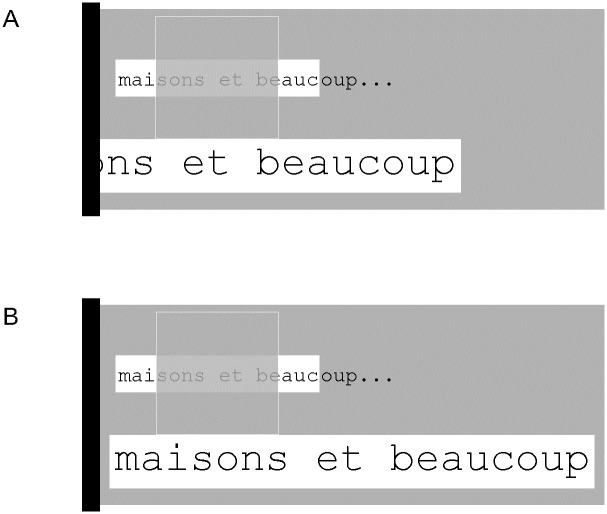
Example of ROAV adaptation to a monitor’s screen border. The grey rectangle represents a portion of the screen delimited by the left screen border (vertical black bar). A/ this shows what would happen if the ROAV location was determined without taking into account the presence of the screen border: the first five letters of the word "maisons" would be invisible. B/ To allow full visibility of the ROAV, the mapping rule adjusts the ROAV location by aligning its left border with the left screen border.

**Fig 7:**
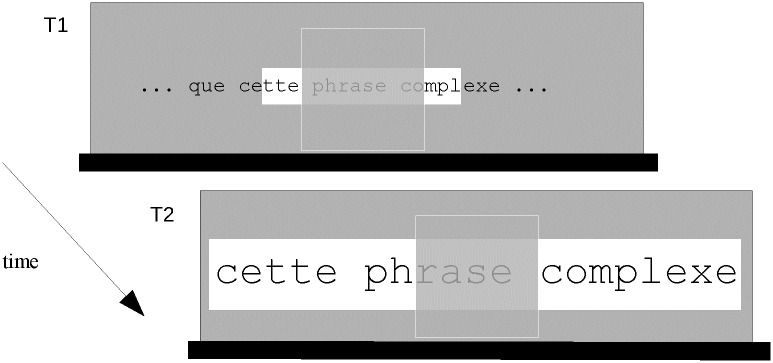
Example of ROAV adaptation to a monitor’s screen border across time. The grey rectangle represents a portion of the screen delimited by the bottom screen border (horizontal black bar). T1/ Same case as in Fig 4 except that the scotoma is very close to the bottom of the screen. T2/ After the button press, displaying the ROAV below the scotoma would make it invisible. Therefore, in this case, the ROAV is displayed with its center aligned with the scotoma's center.

In the previous examples, the location of the ROAV is always determined by aligning its horizontal center with the horizontal center of the scotoma. However, in some cases, this behavior is not desired. For instance, Fig 6A shows that the proximity of the left screen border (black bar) would make it impossible to see the first ROAV letters if this rule was blindly applied. The actual ROI/ROAV mapping rules take this problem into account as illustrated in Fig 6B. Here, the ROAV is entirely visible because its left border has been aligned with the left screen border. These rules apply more generally whenever it is necessary to avoid disappearance of a part of the ROAV as illustrated in Fig 7. Here, the ROAV cannot be displaced below the scotoma, so that its location is determined by aligning its center and the scotoma's center (note that the choice of this location is arbitrary as the ROAV could have been remapped above the scotoma).

### 2.2 - Methods for validation of the gaze-controlled visual aid (condition 1)

The gaze-controlled system (condition 1) was compared with two CCTV-like conditions (condition 2 and condition 3) that allowed a rapid and user-friendly change of magnification level at any moment during reading. The two latter interfaces were inspired by commercially available EVES (CCTV magnifiers) that we improved to facilitate and encourage the alternation between different magnification levels. To allow direct comparisons, the three systems were implemented on the same experimental setup with stimuli displayed on the same screen. Systems 2 and 3 aimed at simulating a standard CCTV magnifier and improving its interface. This simulation was implemented on a standard computer where text stored in the computer could be displaced and zoomed either with a mouse or with a response box.

Examples of three conditions are provided in the *Supporting Information* (gaze-contingent visual aid, condition 1, "S1 Movie"; improved CCTV, condition 2, "S2 Movie"; and CCTV with zoom-induced text reformatting, condition 3, "S3 Movie").

**Fig 8:**
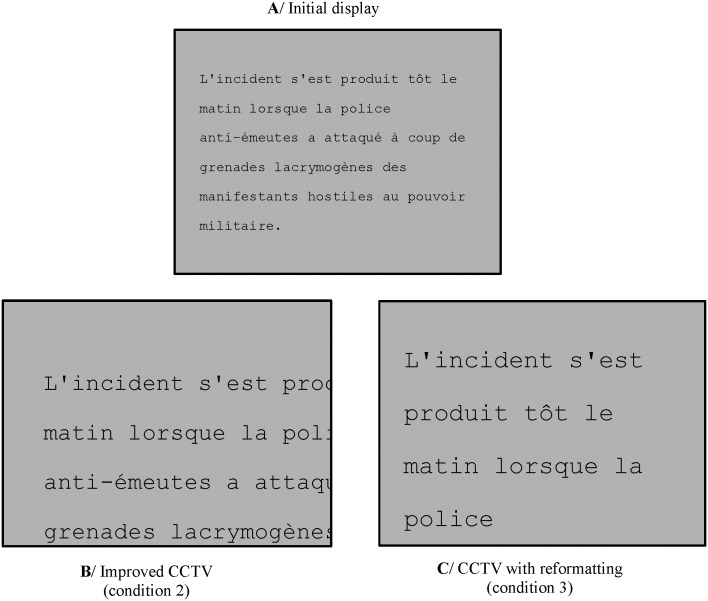
Illustration of the two CCTV-like conditions. The gray rectangle corresponds to the screen’s viewing area. A/ example of a sentence displayed at the beginning of a trial. The full sentence is visible. B/ Effect of magnification in condition 2. The first sentence’s word (“L’incident”) has been maintained visible in the top left screen corner thanks to panning. Note the disappearance and mutilation of words displayed on the right in the initial display. C/ Effect of magnification in condition 3 (same zooming level as in B/). The first sentence’s word (“L’incident”) has also been maintained in the top left screen corner. Note that all the consecutive words of the sentence (up to “police”) are visible thanks to reformatting.

Condition 2 (improved standard CCTV condition) ameliorates standard CCTVs in two ways: a/ by moving a mouse (rather than an x-y platform) in order to pan the text, and b/ by using the mouse scroll-wheel (rather than a button located relatively far from the platform) to zoom the text (Fig 8B). The zooming characteristics in this system are the same as those used in a standard CCTV. Notably, the zooming center always coincides with the monitor's center. One consequence is that the magnification process induces a radial motion of all the words towards the screen borders and consequently a disappearance of all the words close to the four screen’s borders.

Condition 3 (CCTV with zoom-induced text reformatting) was similar to the second one except that text was reformatted in order to fit horizontally within the monitor’s width whenever the magnification level was changed (Fig 8C). This reformatting is actually what happens in any word processing software when print size is changed. This possibility has been implemented in a commercially available CCTV device ("column" layout of the myReader2 video magnifier from Humanware) and implies Optical Character Recognition (OCR) before the system can work. In our system, the text to be read is already stored in ascii format in the computer and subjects interact with the text displayed on the computer monitor by pressing one the four outer buttons of a 5-button response box (RESPONSEPixx Handheld—VPixx Technologies). The left and right buttons are respectively used to zoom in and out. The top and bottom buttons are used to move text in the vertical direction. In terms of navigational constraints, the main difference between conditions 2 and 3 is that horizontal panning is not necessary in the third condition.

#### Subjects

Ten healthy subjects (5 males), who were not aware of the goals of our study, participated in this work (mean age: 24.3 years old; min: 19; max: 27). They all had a graduate degree (from 2 to 7 years after bachelor degree). They had either normal (8 subjects) or corrected-to-normal vision (2 subjects with mild myopia). Subjects were recruited from January to June 2012 and they received either monetary compensation or class credit for their participation. The study was approved by the University of Aix-Marseille ethical review board and was performed in accordance with the Declaration of Helsinki. All participants gave written informed consent according to the guidelines of the University of Aix-Marseille ethical review board prior to their inclusion in the present study.

#### Stimuli

Sentences with black characters were displayed in Courier font, a fixed-width font, with double interline spacing. Sentences were displayed within a virtual box (centered in the middle of the screen) whose width and height were respectively 43° and 30°. This was the size of the initial image displayed at the beginning of any trial, i.e. before any modification induced by subjects. Text was left aligned inside the virtual box as for instance in Crossland and Rubin (2006)[37]. At the beginning of each trial (i.e. before any zooming induced by subjects—Fig 8A), print size was 1°, the value of word acuity at an eccentricity of 6°, a value slightly larger than scotoma's radius [38]. Print size was defined as the visual angle in degrees subtended by a lowercase ‘x’ (x-height) [39].

A text containing one or several sentences, extracted from articles in the French newspapers "le Monde", was displayed on each trial. The original order of the sentences in the article was maintained across trials to increase subjects' motivation to read. The exact number of sentences within each trial was defined by filling the virtual box with as many sentences as possible without splitting the last (or unique) sentence.

#### Apparatus

Stimuli were displayed on a 21-in. CRT color monitor (GDM-F520, Sony, Japan) with a refresh rate of 100 Hz. Mean luminance of the grey background was 86.2 cd/m². At the viewing distance of 40 cm, the display area of the monitor subtended 51° X 38.3° (1024 X 768 pixels); the “° “symbol refers to degrees of visual angle here and throughout the text. Subjects sat in a reclining chair with their eyes at a distance of 40 cm from the monitor. Their neck was comfortably maintained by a custom-built foam restraint fixed on the chair to minimize head movements. This restraint was adjusted so that it was not in contact with any part of the eyetracker. Subjects viewed the screen with their dominant eye while wearing a patch over the contralateral eye. If the latter eye had not been patched, subjects could have closed (consciously or not) their tracked eye, thus stopping the display of the gaze-contingent scotoma and allowing the contralateral eye to look at the scene without any scotoma. The room was dimly lit.

The monitor was driven by a personal computer (referred to as the ‘‘display computer”) that was running a custom software that we developed with OpenGL and with the PsychoPy library [40,41]. Our custom software also used python functions from the Pylink library provided by SR Research to interact with the eyetracker.

#### Eye recording and gaze-contingent scotoma

Subjects’ gaze location (along with other eye data) was recorded 500 times per second with an EyeLink II eye tracker (EL II–head-mounted binocular eyetracker–SR Research Ltd., Mississauga, Ontario, Canada) using the head compensation mode. In this mode, the head is free to move within +/- 30° without altering gaze location recording. Eye location was estimated from pupil centroid. The eye tracker was controlled by a Dimension 4700 DELL PC (referred to as the ‘‘Host computer”). Before each experimental block, a 5-point gaze calibration was performed followed by a 5-point validation (left, middle, right, up and down locations). Calibration and/or validation were repeated until the validation error was smaller than 1° on average and smaller than 1.5° for the worst point.

The experimental program, run on the display computer, interacts with the host computer via a high-speed Ethernet link. This connection allows online processing of eye data and gaze-contingent visual stimulation. In order to simulate an artificial macular scotoma, the program measures gaze location and then displays a mask on the monitor at this location [42,43]. In order to improve the spatio-temporal accuracy of our gaze-contingent scotoma [44–46], we used the rules that we described previously [44]. The gaze-contingent scotoma was a 10° square mask whose color (grey) and luminance were the same as those of the background and with invisible borders.

#### Statistical analyses

Statistical analyses were based on linear mixed-effects models specifying subjects as random factors [47,48]. We used the lmer function—lme4 package [49]—in the R system for statistical computing [50]. We also used the following additional packages for data processing, graphs and tables: ggplot2, dplyr and stargazer. The Akaike Information Criterion (AIC) and likelihood-ratio tests were used to assess an optimal random-effects structure [48]. Likelihood-ratio tests were performed with the anova () function in the lme4 package. In a second step, the significance of fixed effects in the model was assessed in the following way. As the number of degrees of freedom for the t-values of the fixed effects are not exactly known with mixed-effects models, different approximations have been proposed [47,49,51]. However, given the large number of observations in the present study, the t-distribution converges to a normal distribution. Therefore, following standard recommendations, t-values (i.e estimate/standard error) that were larger than 2 in terms of their absolute value were considered as significant–corresponding to a significance level of 5% in a two-tailed test [47,52]. To complement this approach [53–55], we also calculated confidence intervals for the fixed-effects estimates [49]. All these values are reported in relevant tables. Assumptions underlying the models were visually checked with diagnostic plots of residuals [51,56].

#### Procedures

On each trial, subjects were instructed to read the text out loud as quickly as they could without making errors and with the goal of understanding the text [57]. Timing started at the instant the text was displayed on the screen—this was triggered by a subject button-press. Subjects then pressed the same button (this stopped the timing and removed the text) when they had read the last word. Subjects were allowed to spell out proper nouns that they did not know and/or that they found difficult to pronounce. None of the texts was read more than once by any subject. To remind subjects that accuracy and comprehension were important, visual feedback was provided at the end of each trial to indicate the number of words that had not been read correctly. In addition, subjects had to orally answer comprehension questions asked by the experimenter every 10 texts on average. If at least one word in the text was read incorrectly, the text was judged as incorrect and excluded from statistical analysis. Reading speed was calculated in ‘‘standard-length words” per minute where each six characters counts as one standard-length word [58].

Each subject performed one training session and 8 experimental sessions (each lasting about 1 hour and performed on different days). These sessions were always performed within three consecutive weeks (except for subject TD for whom the total period was 5 weeks). The training session allowed subjects to get used a/ to reading with a scotoma and b/ to using the three visual aids. This session also allowed us to determine the location that subjects preferred in order to display the ROAV with respect to their scotoma. This was achieved by using a gaze-contingent hemi-field scotoma forcing subjects to use eccentric vision (beyond 5° eccentricity) either in the upper or lower visual hemi-field [59,60]. Ten one-line sentences were read with an upper hemi-field and ten one-line sentences were read with a lower hemi-field. After reading these sentences, subjects were asked to report their preference: results showed that all observed preferred to read with their lower visual hemi-field. The ROAV was therefore displayed for each subject below the scotoma in the experimental sessions. Each experimental session contained the three different conditions of visual aid that were run in randomly interleaved separate blocks. The number of blocks for each session was not specified in advance: subjects with high reading speeds could read more sentences within each one-hour session than slow readers.

## 3 - Results

### 3.1 – Implementation of the gaze-controlled system (condition 1)

The gaze-controlled enhanced vision system that was eventually implemented and tested in the present work can be appreciated by watching the movie in the *Supporting Information* (S1 Movie). It is based on the following key principles:

1. At any moment, the area that can be potentially enhanced (here magnified) is relatively small compared to the screen: this is the Region Of Interest (ROI). The ROI is defined here as a group of adjacent letters. This definition implies some sort of figure/ground segmentation process and thus necessitates either low-level image processing and/or Optical Character Recognition. We believe that initial segmentation of an “intelligent” ROI is an essential feature of our system.
2. The ROI’s location is controlled by the subject (here through gaze) and a visual feedback (highlighting) is constantly provided as to which ROI is currently selected [33].
3. The visual magnification is triggered by a manual control so that subjects decide online whether or not they want to apply it. This is to allow subjects to have full control over the triggering of the visual aid and thus avoid the Midas touch problem [32,61]. The Midas touch problem is a fundamental difficulty faced by any gaze-controlled system: at any moment, the system must decide whether gaze is intended to extract visual information or to activate a specific command. Without this distinction, subjects find that everywhere they look, voluntarily or involuntarily, a new command is triggered.
4. The magnification process is also “smart” in several ways (from Figs 4 to 7). Firstly, the ROI is not necessarily magnified as it is. Instead, a Region Of Augmented Vision (ROAV) is calculated from the ROI before the actual magnification. The magnified ROAV can thus contain a number of letters that is different from that of the ROI. The goal of this adaptation is to provide a magnified region (ROAV) containing only full words (Fig 4). Secondly, the relative location of the ROAV is adapted (depending on the proximity of the screen’s borders) so that it is always fully displayed within the screen.

### 3.2 – Validation of the gaze-controlled system

We first assessed whether and how subjects used the possibilities offered by the three visual aids. For the first condition (gaze-contingent aid), we measured the number of times the button inducing ROAV display was pressed for each trial. We then calculated the ratio between this number and the number of standard-length words, i.e. 6 characters, [58] of the corresponding text. The median value across subjects was 0.45 (1st quartile: 0.26; 3rd quartile: 0.66) indicating that subjects used the aid approximately every two standard-length words. The duration of the ROAV display had a median value of 840 ms (1st quartile: 753; 3rd quartile: 1005) across subjects. The proportion of time spent with the ROAV activated had a median value of 0.33 (1st quartile: 0.15; 3rd quartile: 0.54) across subjects.

**Fig 9:**
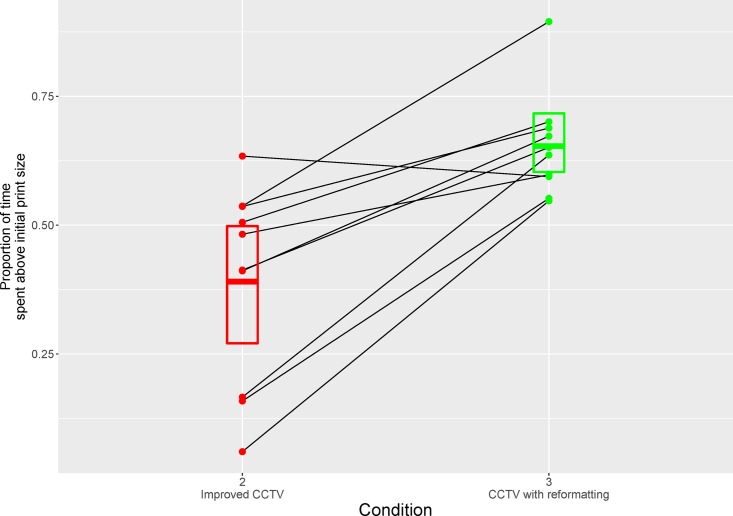
Proportion of time spent with print sizes larger than initial size (see Fig 8A) for the 10 subjects (solid lines) as a function of the two CCTV conditions (2 and 3). Boxes represent mean proportion (middle thick line) and bootstrapped standard errors for each condition.

Then, for the second and third conditions (simulations of CCTV), we measured for each subject a histogram of the time spent for different print sizes (time was normalized with respect to total duration). From these histograms, we calculated the proportion of time spent for print sizes larger than 1.1° (i.e. larger than initial character size). Results plotted in Fig 9 illustrate the dramatic difference between the two conditions. A mixed-effects analysis showed a significant difference between conditions (est = 0.26, t = 4.99, 95%CI = [0.17, 0.37]; transforming the proportions with an arcsine function did not change the pattern of results). The clear and interesting pattern is that using CCTV with reformatting (condition 3) encourages subjects to magnify text more often: in this mode, all subjects spend more than 50% of the time with a magnification level that is above the initially displayed character size. Note that the proportions reported for conditions 2 and 3 cannot be meaningfully compared with the proportions measured in condition 1 (proportion of time spent with the ROAV activated). In condition 1, proportions reflect the need to trigger the magnification enhancement which corresponds to a unique print size. In contrast, in conditions 2 and 3, subjects can use as many print sizes as they wish.

At the end of the experiments, subjects were asked to give ratings for the 3 systems on a comfort scale with a number from 1 to 10 (with 1 being the least comfortable and 10 being the most comfortable). The mean rating for condition 1 was 7.2 and was not significantly different from the rating for condition 3 (CCTV with reformatting). However, mean rating for condition 2 was significantly smaller (Table 1).

**Table 1:** Results of the mixed-effects analysis for the comfort ratings. For each effect, the coefficient’s estimates are accompanied by 95% confidence intervals in parentheses and corresponding t-values are displayed on the next line. Reference level: condition 1 (gaze-controlled aid).

	*Dependent variable*:
	Comfort rating (1–10)
Intercept (cond.1)	7.2
	(6.6, 7.8)
	t = 22.1
Improved CCTV (cond. 2)	-2.1
	(-3.0, -1.2)
	t = -4.6
CCTV with reformatting (cond. 3)	-0.5
	(-1.5, 0.4)
	t = -1.1
Observations	30

Reading speed data were then analysed with mixed-effects model. In the model with the optimal random structure, the slope of the effect of the rank of trials was random across subjects thus allowing for different individual effects of learning or fatigue [47]. The fixed-effects were a/ the type of visual aid, b/ the rank of trials and the interaction between the two effects. The fixed effects of this mixed-effects model are presented in Table 2 (left column). The most important result is that reading performance in condition 1 (the reference level) was not worse than in any of the two other conditions. It was actually significantly higher than in condition 2 (improved CCTV without reformatting) although the size of this effect (about 10%) does not seem important from a clinical perspective. And it was not distinguishable from condition 3. Another important result is that we did not find any significant interaction between the conditions and the effect of the rank of trials: the slope of the rank of trials is similar for the three conditions. These interaction effects were therefore removed from the final model (right column in Table 2) inducing a higher estimate for the effect of the rank of trial (as well as slightly higher accuracy for this effect). Note also the smaller values of the goodness-of-fit indices (bottom lines of Table 2) indicating better fits for the final model.

**Table 2:** Results of the mixed-effects analyses for the fixed effects. For each effect, the coefficient’s estimates are accompanied by 95% confidence intervals in parentheses and corresponding t-values are displayed on the next line. First colum (1) is for the model with the optimal random structure and all the fixed effects. Second column (2) is for the final model, i.e. the same as in the first column but without interaction terms. Reference levels for the analyses are condition 1 (gaze-controlled aid) and mean rank of trials. “:” stands for interaction.

	*Dependent variable*:	
	Reading Speed (natural log)	
	(1)	(2)
Intercept (cond. 1)	3.7437	3.7425
	(3.5873, 3.8996)	(3.5862, 3.8985)
	t = 49.0034	t = 48.9984
Improved CCTV (cond. 2)	-0.1160	-0.1152
	(-0.1661, -0.0659)	(-0.1653, -0.0650)
	t = -4.5368	t = -4.5028
CCTV with reformatting (cond. 3)	-0.0064	-0.0054
	(-0.0546, 0.0419)	(-0.0536, 0.0429)
	t = -0.2585	t = -0.2174
Rank of trial	0.0022	0.0026
	(0.0003, 0.0041)	(0.0008, 0.0045)
	t = 2.2812	t = 2.9849
C2:Rank of trial	0.0011	
	(-0.0001, 0.0022)	
	t = 1.7735	
C3:Rank of trial	0.0004	
	(-0.0007, 0.0016)	
	t = 0.7367	
Observations	1165	1165
Log Likelihood	-478.6683	-467.0893
Akaike Inf. Crit.	977.3366	950.1786
Bayesian Inf. Crit.	1027.9410	990.6624

**Fig 10:**
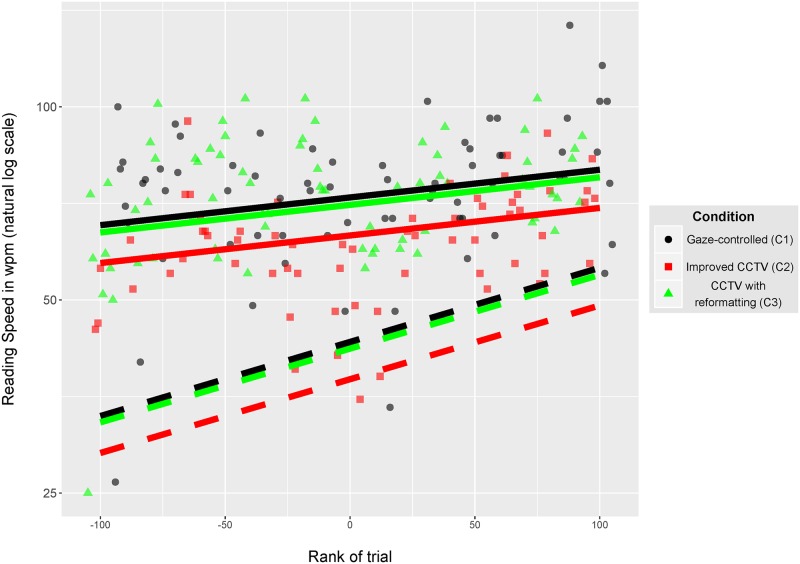
Results for subject ‘CHAR’ who had the highest reading speed mean. Symbols show reading speed (note the natural log scale) as a function of the rank of trials and for the 3 conditions (condition 1: black; condition2: red; condition 3: green). Fixed-effects are shown with dashed lines whereas the estimated random effects for this subject (i.e. the conditional means) are shown with solid lines. For visual clarity, an artificial vertical jitter was added between the lines of conditions 1 and 3 to avoid overlapping.

Reading speed data for each trial are shown with symbols in Fig 10 for the subject with the highest intercept value (subject 'CHAR'): reading speed is plotted as a function of the rank of trials and for the 3 conditions. The fixed-effects from the final mixed-effects model (right column in Table 2), which represent the effects at the population level, are represented in Fig 10 with dashed lines. The estimated random effects for this subject (i.e. the conditional means) are shown with solid lines. They are above the fixed-effects lines and their slopes show that this subject had a moderate learning effect over the 9 hours of experiments when compared with the population effect. The reading speed values observed at the end of the experiment are close to 80 words per minute, a high value that can be considered as indicating “fluent” reading for low vision readers [17].

**Fig 11:**
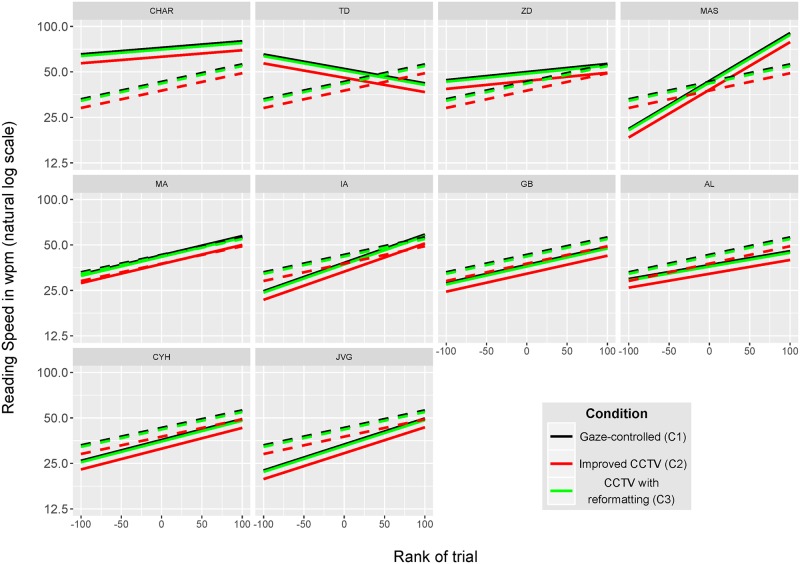
Fixed-effects and random effects from the final mixed-effects model for all subjects. Characteristics and layout of this figure are the same as in Fig 10.

Fig 11 offers a graphical summary of all individual random effects (conditional means) estimated from the final mixed-effects model with the same color codes as in Fig 10. The subject with highest reading speed (“CHAR”) is shown in the top left plot. The other plots are ordered in decreasing order based on the individual intercept reading speeds (from top left to bottom right). In contrast to subject “CHAR”, most subjects have a low reading performance which corresponds to a “spot reading level” [17]. All subjects, except one (“TD”), have estimated random effects with positive slopes indicating overall significant learning. One subject (“MAS”) shows an impressive increase of reading speed by a factor close to 4. At the population level, the fixed effect of the rank of trial has a slope indicating a 30% increase in reading speed over 100 trials.

## 4 - Discussion

This proof-of-concept study aimed at evaluating the feasibility of a gaze-controlled visual aid specifically designed for people reading with Central Field Loss (mostly AMD patients). In the present work, Central Field Loss was artificially induced in normally-sighted subjects with a gaze-contingent simulated scotoma (size: 10°). The key principles underlying this new aid are summarized in a flowchart in Fig 1 and were implemented in a standard computer.

The effective use of this system by patients with CFL relies on one main assumption. Patients should be able to use a Preferred Retinal Locus (PRL) in order to fixate a static target [62,63]. This is necessary to allow patients to correctly perform the calibration of the eyetracker at the beginning of experiments (calibration consists in successively fixating static dots scattered across the monitor). Most patients develop such a “fixation”PRL with different time scales [64]. Note that it is still debated whether patients only use this “fixation” PRL or if they use different PRLs when they read text [65–67]. This unsettled issue is not a problem for our system as subjects can explore the ROAV with several eye movements and use one or several PRLs as they wish.

Thus, the main open question which should be a topic for future research concerns the most optimal ways of controlling the ROI with gaze. Research should also establish whether patients should be allowed to choose which kind of gaze/ROI link they prefer. In the present implementation of our system, we chose to use the scotoma’s location to control the ROI because our normally-sigted subjects reported in preliminary experiments that this was the most comfortable option. However, it is possible that this might be different with patients who have used a PRL for a long time. They might possibly prefer to use their “fixation” PRL in order to control the ROI during the reading process. This question is currently investigated in our team.

Our gaze-contingent enhanced vision system is inspired by some important features that have been previously proposed for normally-sighted subjects (e.g. the ‘magic’ or ‘smart’ lens concept), mainly in the human-machine interface literature in the context of zoomable user interfaces (ZUI) with or without gaze control [31–33,68–74]. The idea that gaze-contingent visual enhancement might be used specifically to aid subjects with scotomas has already been proposed [75–79]. The main idea in the proposed algorithms was a “remapping” process that was able to continuously warp and redistribute information being hidden by the scotoma to the still functioning parts of the retina. However, testing these algorithms with three patients having CFL in a reading task produced disappointing results [76]. Informal observation of these "continuous remapping" algorithms in our laboratory suggested to us that the main problem relied on the locations of words that were constantly changing on the screen as the eyes moved, whether these movements were voluntary or not. Subjects reported that words were continuously jumping around so that it was very difficult to integrate information across successive fixations, especially when they tried to fixate a single word. This continuous gaze-induced remapping does not occur in our system as any ROAV (once displayed by a button press) is fixed on the screen. The ROAV thus remains motionless on the screen, whether the eyes move or not, until subjects release the button.

In summary, the development of our gaze-controlled system was guided by both successes and failures of previous studies. Our main goal was to build a system allowing subjects with CFL to alternate easily between a global view of the text and a magnified view of words [28]. Importantly, even during magnification of words (i.e. ROAV display), a significant global view of the text was preserved, thus facilitating navigation in the page [eg. 23–25]. In addition, this ROAV was fixed on the screen until its perceptual identification was achieved i.e. until the subject released the button.

This system (condition 1) was compared with two other systems (conditions 2 and 3) whose principles were based on commercially available CCTVs and implemented on the same computer as in condition 1. In the CCTV-like conditions (2 and 3), it was possible to alternate between global presentation of the text and magnification of the whole text (Fig 8). However, it was impossible to obtain the simultaneous view of global and locally-magnified information as offered in condition 1.

The first important result is that subjects did often alternate between the global and local magnification views offered in the gaze-controlled condition. This is not a trivial result because allowing subjects to modify magnification during reading, even with a user-friendly interface, does not guarantee that they will exploit this possibility. It seems therefore that subjects did not find this possibility either too cumbersome or not helpful. This was also true in the two CCTV-like conditions (2 and 3) with an additional interesting result illustrated in Fig 9. On average, subjects spent much more time reading with large magnification levels in condition 3 (CCTV with reformatting) than in condition 2 where reformatting was absent (Fig 8). It seems therefore that reading text with all successive words present on adjacent lines is much preferred by subjects, presumably because it significantly reduces the page navigation problem.

This preference for the reformatting mode (cond. 3) is however not accompanied by a difference in reading speed thus suggesting that the preference is related to reading comfort. This is consistent with the comfort ratings showing that conditions 1 and 3 were rated equally whereas condition 2 was rated with a significantly smaller grade (Table 1). It would be interesting to test in future research if this kind of preference would be reflected by more psychological constructs such as motivation to read or by measures such as productivity, i.e. the time during which subjects manage to read continuously. This kind of productivity difference was observed in a study where only 4.5% of subjects using an optical device were able to read continuously for 40 minutes, while 59.1% of the subjects using handheld CCTVs and 72.7% of the subjects using stand-mounted CCTVs were able to read the full 40 minutes by the end of training [80]. A final important note concerning whether subjects used the magnification modifications is that we made every effort to ensure that these modifications were as user-friendly as possible in the two CCTV-like conditions. Our goal was conservative in that we did not want to create a disadvantage that might have been induced by a poor human-machine interface (cf. the inconvenient placement of the knobs that control magnification in several commercial CCTV systems).

In terms of reading speed, our results show a modest advantage (about 10%) for the gaze-controlled system with respect to the improved CCTV (condition 2) and no difference when compared with the CCTV with reformatting system (condition 3). This result is very encouraging essentially because it shows that the gaze-controlled system fares well with the two other conditions which are considered as the most advanced commercial EVES. In this respect, the absence of interaction between the conditions and the effect of the rank of trials is also important as it shows that learning in condition 1 does not present any specific problems compared to the two CCTV-like conditions.

These encouraging results suggest that the principles implemented in condition 1 have the potential to be improved and extended in different directions. Several features might be considered. In the present study, the enhancement was simply a magnification with a constant level. However, it would probably be helpful to add the possibility of letting subjects use different levels of magnification (up to screen’s size) while the ROAV is displayed. In the case of large levels of magnification, another option to be investigated would be the possibility to adapt the size and format of the ROAV to the size and shape of the display. For instance, with a very large monitor, it would be interesting to study if displaying an ROAV containing two or three lines of text is more efficient than using a single line. And even more sophisticated enhancements might be added. For instance, digital filtering tailored to low vision constraints might be applied within the ROAV [81]. Some low-level visual modifications could easily be implemented within the ROAV with the goal of reducing crowding [82]. For instance, a smooth transition between the ROI and the ROAV might be used instead of an abrupt displacement, which could potentially reduce crowding [83]. Even higher-level modifications induced by automatic text simplification might also be envisaged [29,84,85]: thus, some complex words within an ROAV might be replaced by lower complexity synonyms. The possibilities offered by digital image processing combined with text processing (once OCR has been performed) are actually endless and should be guided by theoretical results concerning our knowledge of limiting factors in low vision reading [15]. In sum, we believe that our condition 1 offers a basic algorithm that could be improved in several ways in order to increase perceptual stability, comfort and overall reading performance.

In addition, the ideas underlying condition 1 might be implemented in very different kinds of setups. For instance, if patients want to use a CCTV-like system, i.e. at a relatively short viewing distance, then gaze-control could be achieved with a remote eyetracker integrated within the CCTV. This kind of setup would remove the constraint of wearing special glasses or helmets that include an eyetracker. Ideally, helpful options already present on CCTVs thanks to text digitalization, such as text reformatting (cf. condition 3), should be combined with our system. Another example concerns the use of new fonts able to improve reading performance [86]. The huge potential of EVES is precisely the ability to additively combine several helpful sources whose benefits are modest when taken individually. Another interesting option with a short viewing distance is that gaze-control could be replaced by a haptic interface. Patients could then select ROIs by pointing at some locations on a touch screen. This could be implemented for instance on tablet computers or e-books readers [87]. Apart from the interface difference (gaze vs. touch), all other features of condition 1 would remain the same. It is difficult to predict if this option would be found comfortable by patients as this would imply many hand pointing movements, but this is an option that would be technically easier to implement.

In addition, it would seem promising to implement the gaze-controlled system in see-through glasses [35]. One of the most promising and challenging future uses of head-mounted displays is in applications in which virtual environments enhance rather than replace real environments [34]. This is referred to as augmented or enhanced reality [88,89]. Convincing evidence suggests that augmented reality has a huge potential to help low vision patients [36,90–93]. In this kind of setup, an important asset of gaze control is that subjects can interact with distant displays (signs in a street or large screens at home) even though using a mouse is not possible [31,32].

## 5 - Conclusion

Future research should establish whether low vision patients can learn to efficiently use a gaze-controlled system based on the principles developed in condition 1. Reasons to be optimistic about adaptation abilities of patients rely on converging evidence that perceptual learning processes are still functional with Central Field Loss reading [94–97]. In addition, there is evidence that cortical reorganization processes are active in low vision patients following long-term adaptation to scotomas [98].

## Supporting information

S1 Movie Example movie of the gaze-controlled visual aid (condition 1). (MP4)

S2 Movie Example movie of the improved CCTV (condition 2). (MP4)

S3 Movie Example movie of the CCTV with reformatting (condition 3). (MP4)
